# P102 Ro 52 and anti centromere positive Sjögren’s disease with myocarditis and MALT lymphoma

**DOI:** 10.1093/rap/rkag113.132

**Published:** 2026-11-03

**Authors:** Kriti Sharma, Azeem Ahmed, Marvi Iftikhar, Urwah Ahmed, Antony Psarras, Joel David, Shirish Dubey

**Affiliations:** Oxford University Hospitals NHS Foundation Trust, Oxford, United Kingdom; Oxford University Hospitals NHS Foundation Trust, Oxford, United Kingdom; Oxford University Hospitals NHS Foundation Trust, Oxford, United Kingdom; Oxford University Hospitals NHS Foundation Trust, Oxford, United Kingdom; Oxford University Hospitals NHS Foundation Trust, Oxford, United Kingdom; Oxford University Hospitals NHS Foundation Trust, Oxford, United Kingdom; Oxford University Hospitals NHS Foundation Trust, Oxford, United Kingdom

## Abstract

**Introduction:**

Sjögren’s disease (SjD) typically presents with sicca symptoms but can also present with extra-glandular features affecting the skin, lungs, kidneys, nerves and, rarely, the heart, typically as heart blocks. Commonly associated antibodies are Antinuclear (ANA), Anti-Ro and Anti-La. We describe a 74-year-old woman with longstanding sicca symptoms who developed MALT lymphoma in 2014, after which a diagnosis of SjD was suspected and eventually confirmed in 2022 with ANA, Anti-Ro 52 and Anticentromere (ACA) antibodies. After a decade her disease became complicated by recent development of myocarditis.

**Case description:**

A 74-year-old woman of Southern European ancestry with biopsy and antibody proven SjD diagnosed in 2022. She was treated with hydroxychloroquine, colchicine (SjD-related pleural effusion), and previously with methotrexate (2023-2024) and rituximab (2g in 2024). She had Raynaud’s, localised submandibular MALT lymphoma resected in 2014, renal cell carcinoma with partial nephrectomy in 2015, previous myocardial infarction requiring coronary stenting, biliary duct dilation and ongoing heavy smoking.

She presented in 2026 with a 2-year history of un-intentional weight loss of 14 kg, and over six weeks progressive limb weakness, paraesthesia, dysphagia, vomiting, diarrhoea and cold peripheries, and increased urinary frequency prompting admission.

Examination showed proximal limb weakness, reduced lower limb sensation and cold peripheries, without sclerodactyly, microstomia, digital ulceration or telangiectasia. CRP was 36mg/L at admission, falling spontaneously to 9.1mg/L within a week, felt to reflect a concurrent E. coli urinary tract infection. Immunology reported positive anti-Ro52, ACA and ANCA (MPO/PR3 negative), negative RF, dsDNA, anti-HMGCR and myositis antibodies. Complements and Immunoglobulins were normal. Nerve conduction studies showed length-dependent sensory axonal neuropathy. Imaging showed oesophageal, colonic mural oedema and a right lung lower lobe cystic lesion.

During admission she developed chest pain. Troponin-I peaked at 973ng/L and ECG changes of NSTEMI. Anti-platelets were started but coronary CT excluded flow-limiting disease. Echocardiography showed Ejection Fraction 30–35% with apical akinesia; cardiac MRI showed Ejection Fraction 49% with basal inferior and anterior wall hypokinesia with widespread myocardial oedema but without amyloidosis; NT-proBNP peaked at 37,525ng/L.

Endoscopy, colonoscopy and PET-CT excluded malignancy. Barrett’s oesophagus was identified.

SjD-myocarditis was diagnosed. Treatment included mycophenolate mofetil (stopped due to diarrhoea after 2 weeks), followed by prednisolone 30mg daily (tapering regimen) along with monthly intravenous cyclophosphamide 500mg with antimicrobial and entecavir prophylaxis (hepatitis B core antibody positive, PCR negative). She has improved clinically and biochemically. Treatment is ongoing.

**Discussion:**

This case highlights an under-recognised aspect of SjD-myocarditis. Autoimmune myocarditis is an infrequent cause of cardiac involvement in autoimmune rheumatic disease, occurring more often in SLE than in SjD, where only a handful of cases have been reported. Emerging case reports describe SjD-myocarditis presenting acutely, at times with cardiogenic shock, with diagnosis supported by cardiac MRI meeting Lake Louise criteria and marked improvement following high-dose corticosteroids. Our lady’s markedly elevated troponin and NT-proBNP, and diffuse myocardial oedema on MRI in the absence of flow-limiting coronary disease are consistent with this rare phenomenon. The discordance between her echocardiographic (30–35%) and MRI-derived (49%) ejection fractions illustrates a recognised limitation of echocardiography in this context; MRI, with its ability to characterise myocardial tissue and track serial change, was considered more specific and guided decisions.

Anticentromere positivity in SjD, even without cutaneous features of limited systemic sclerosis, should prompt screening for systemic sclerosis-spectrum organ involvement, as in this patient who lacked sclerodactyly.

Biliary duct dilation does raise suspicion of associated primary biliary cholangitis however this was previously ruled out.

A central management challenge was distinguishing inflammatory extra- glandular disease from recurrent or new malignancy, given her oncological history, requiring systematic exclusion via endoscopy, colonoscopy and PET-CT before immunosuppression could be safely escalated.

Treatment with mycophenolate (though not tolerated by her), corticosteroids and cyclophosphamide, chosen for organ-threatening cardiac disease, mirrors approach reported in comparable cases of SjD-myocarditis where immunosuppression achieved clinical improvement.

**Key learning points:**

• Cardiac involvement, including myocarditis, is a rare but serious extra-glandular manifestation of SjD and should be considered in patients with unexplained troponin rise or heart failure and elevated inflammatory markers.

• ACA positivity in SjD should prompt consideration of overlap with limited systemic sclerosis, even without sclerodactyly or Raynaud’s phenomenon

• In patients with a significant prior malignancy history, such as MALT lymphoma or renal cell carcinoma, new multi-system inflammatory symptoms warrant thorough exclusion of malignancy or recurrence before committing to immunosuppression, even when the overall picture strongly favours autoimmune disease.

• Complex, multi-organ presentations of SjD require coordinated multidisciplinary input, including rheumatology, cardiology, gastroenterology, respiratory medicine and haematology, to sequence investigations and treatment safely.

• Cyclophosphamide, with appropriate anti-microbial and antiviral prophylaxis, is a reasonable option for organ- or life-threatening SjD-related inflammation such as myocarditis, once malignancy has been excluded.

• Standard serological markers, including C-Reactive Protein, correlated poorly with underlying disease activity in this case–the admission C-Reactive Protein rise reflected a concurrent urinary tract infection rather than SjD itself–so clinical and imaging findings should guide treatment urgency rather than acute-phase reactants alone.

